# Quality of Information for Skin Cancer Prevention: A Quantitative Evaluation of Internet Offerings

**DOI:** 10.3390/healthcare9020229

**Published:** 2021-02-19

**Authors:** Wolfgang Uter, Christina Eversbusch, Olaf Gefeller, Annette Pfahlberg

**Affiliations:** Department of Medical Informatics, Biometry and Epidemiology, University of Erlangen/Nürnberg, 91054 Erlangen, Germany; christina.eversbusch@fau.de (C.E.); olaf.gefeller@fau.de (O.G.); annette.pfahlberg@fau.de (A.P.)

**Keywords:** skin cancer, prevention, information, internet, DISCERN, HONcode

## Abstract

(1) Background: Different sources of information are used by the population regarding skin cancer prevention. The pertinent quality of information that can be retrieved via an internet search engine needs assessment; (2) Methods: Four topical searches in common language were entered into Google™. The first 200 “hits” were stored for further use. Eligible websites were evaluated using content-based criteria based on the current German medical guideline “Skin cancer prevention” and employing generic (DISCERN, HONcode) quality criteria. (3) Results: Overlap between the four search results was between 0 and 7 of 200. The completeness of relevant content was scored with a median of 10 points (first quartile (Q1):6; Q3:14) and thus, it was much lower than the theoretical maximum of 43 points. Global quality, with a maximum of 10 points, was 3 in median (Q1:2; Q3:4). Quality and completeness, respectively, were somewhat higher in the higher ranks of search results. The generic quality was moderate. (4) Conclusions: A direct comparison with other sources of information (print, audio-visual, presentation, or personal counselling) is not possible, but important deficits concerning the quality and scope of relevant information on the internet are demonstrated.

## 1. Introduction

The incidence of malignant melanoma continues to rise worldwide [1,2], albeit at least in Germany at a slower rate in the last decade [3]. In Germany, the crude incidence rate in 2016 has reached 26.7/100,000 in women and 29.8/100,000 in men; for non-melanoma skin cancer, the incidence is higher by a factor of at least 10 [4]. In addition to constitution, chemicals, and heavy metals [5], the eminent role of ultraviolet (UV) radiation, whether of solar or artificial origin, in the development of both malignant melanoma and non-melanotic skin cancer (squamous cell carcinoma or basal cell carcinoma) is well established; the International Agency for Research on Cancer (IARC) has classified UV radiation as a class 1 human carcinogen in 2009 [6]. The consequences of climate change could further increase UV exposure in Germany [7] and other countries subjected to similar changes. Thus, it is evident that UV avoidance or reduction is crucial for skin cancer prevention, as is shown, for example, in the recommendations on the contents of an informative medical consultation in the current German medical guideline “Skin cancer prevention” [8], which is on the highest level of methodological rigor termed “S3” in Germany [9].

However, optimum sun protection does not yet seem to have been fully achieved, at least not in everyday life in Germany. Indeed, empirical data from Germany in terms of the standardized telephone survey of a representative population sample (*n* = 3000; National Cancer Aid Monitoring on Sunbed Use (NCAM) study) of 14 to 45-year-olds in 2015 showed shortcomings above all among men, people with a low level of formal education, and people with a migration background [10]. In a subsequent wave of the survey and using the same approach, the practice of using sunscreen products was reviewed in 2018. It was found that although almost 80% of the respondents self-reported to use sunscreen products, most of them did not follow the recommendations for optimal application [11], which is possibly in connection with an over-optimistic assessment of their own probability of developing skin cancer [12]. With regard to artificial UV radiation, which is almost synonymous with visiting sunbeds, the proportion of sunbed users in Germany decreased from 11% of the above-mentioned respondents in the NCAM study in 2015 to 8.8% in 2018, but there was a worrying increase in the number of minors, despite the ban on sunbed use for this age group [13].

Repeated surveys have also been conducted with parents of children who are in a nursery school. These demonstrated relatively good knowledge about skin cancer risk factors [14,15]. By contrast, a lack of translation into adequate preventive behavior, which is additionally modified by attitudes and opinions was seen [16,17]. This is particularly true in everyday settings, compared with the prototypical beach holiday [18], and it is aggravated by the absence of shading for children’s playgrounds [19]. As one of the above-mentioned surveys, a sample of 3129 families with children attending a kindergarten in Franconia was surveyed between October 2011 and February 2012 by means of a self-administered questionnaire. This also addressed the question of sources of information on sun protection and skin cancer prevention the parents would use. While the continued importance of traditional media such as print media, radio/TV as well as lectures or counseling was evident, an appreciable proportion of 13.3% (also) obtained their information from the internet [19]. Unexpectedly, when analyzing the association between the source of information and the quality of knowledge, internet use was associated with poorer knowledge compared to the above-mentioned “classical” sources [20].

Hence, the question arose as to whether a possibly poor quality of information on the internet could be the cause of the poorer knowledge. This hypothesis is the starting point of the present study, in which both the specific content quality of information offered on sun protection or skin cancer prevention on the Internet and more general, generic aspects of the reliability and credibility of health information were empirically investigated for the first time.

## 2. Materials and Methods

In order to achieve the highest possible level of representativeness, Google™ was used as the search engine, which has a market share of 92% of Internet search engines in Germany [21]. To narrow down the numerous potential search terms, a preliminary study asked 49 people of different ages and background to suggest meaningful keywords for a search for information on skin cancer prevention. The most frequently mentioned terms with more than ten mentions were sunburn, sun protection, sun protection factor, and sun cream. Four search queries were formulated based on these mentions (originally in German): A, “Skin cancer sun protection”; B, “How to protect yourself from the sun”; C, “Sun protection for children”; and D, “Preventing sunburn”. “Sun protection factor” and “sun cream” were not used to avoid limiting the search results to this subsidiary form of UV protection.

These four search queries were entered one after the other on a private DSL line and a newly installed Firefox browser (version 56.0.2, 32 bit) under Windows™ operating system without ad blocker. Before each new search request, the old Firefox profile was deleted, a new one was created, and a new IP address was assigned to avoid referencing. A private internet access, i.e., outside the academic IP address range, was used. In addition, a web-based “fingerprint” of the site [22] was created and stored to verify the success of the above measures. In order to simulate a typical “click behavior”, a page was clicked on each of the 20 10-hit pages, which had previously been randomly determined by the statistics program R (version 3.4.1, R Foundation for Statistical Computing, Vienna, Austria; RRID:SCR_001905; [23]). The first 200 hits of each search request were saved and listed in a tabular spreadsheet format for further processing. Only web pages that were available without registration and their integral links as part of the web offer were treated further. There was no navigation to sources outside the original website. Commercial web pages, product tests, forums, pages with irrelevant content, and those already recorded were documented as such but not investigated further.

The evaluation of the websites was based on both content quality and completeness, and generic quality criteria. Forty-three criteria were defined based on the list of contents to be discussed in a medical consultation focusing on skin cancer prevention, as laid down in the medical guideline “Skin cancer prevention” [8]; see Table 1. A value of 1 was assigned if the item was mentioned appropriately, and 0 otherwise. By adding up these single points, a content completeness score that could assume values between 0 and 43 was derived. Furthermore, it was recorded whether helpful visual content was available. Finally, a global quality score between 0 and 10 was assigned, independent of the above-mentioned content completeness score, which is similar to a “Physicians Global Assessment” score.

The DISCERN-Score [24] was used as a generic tool to assess the quality of health information. This tool is provided in Germany by the Department of Epidemiology, Social Medicine and Health System Research of the Hannover Medical School and the Medical Central Office for Quality Assurance. DISCERN was initially developed for the evaluation of patient information from the patient’s perspective, especially concerning treatment alternatives, but it can also be used to evaluate internet-based health information. Since treatment alternatives are not relevant in the case of skin cancer prevention, only the first 8 of the total of 16 DISCERN items were used, see Table 2. For these, ordinal values of 1 (“No”), 2 (“Partial”), and 3 (“Yes”) were assigned in three steps, which were added up to form a DISCERN total score. In contrast to the original concept, in which a more refined five-point scale is used, this simplified scale was chosen, since no comparisons with other DISCERN results were intended.

The Health On the Net (HON) foundation is an international non-governmental organization that offers the HONcode certification [25]. HONcode aims at providing an ethical standard for benchmarking the quality of medical information by appraising the efforts of a website to publish transparent information. The transparency of a website increases the ability of its users to verify the objectivity and accuracy of the published data. According to the developers, the HONcode was designed for three target groups: the general public, medical professionals, and website publishers. For the purposes of the present study, it was not checked whether a website was HONcode certified, but the corresponding checklist (see Table 3) was used. For each of the 8 items, three-level ordinal values of 1 (“no/not”), 2 (“partially”), and 3 (“yes/complete”) were assigned. This scaling was chosen in accordance with DISCERN in order to create a comparable range of values. The individual values of the 8 items were added up to arrive at an HONcode total score.

In order to investigate the relationship between the ranking of an identified website in the hit list generated by Google™ and its quality, correlation was quantified using the Spearman correlation coefficient with an accompanying 95% confidence interval (CI; calculated by bootstrap). Moreover, the rankings were categorized into 3 classes, namely the first 10 hits (which are usually displayed together on the first results page), hits 11 to 30, and all subsequent hits. The rankings were based on the original list of 1 to 200. Differences in the distribution of the different score values for quality between the four search queries “A” to “D” were statistically tested by the Kruskal–Wallis test.

The reliability of the assessment of quality and completeness was addressed by two examiners who investigated search query “A” in a mutually blinded fashion. This search query was regarded as representative for the other 3 queries. The degree of agreement of the four respective sum scores (content completeness score, global quality score, and DISCERN and HONcode total scores) was quantified with Spearman correlation coefficients. In case of the three-level individual items of DISCERN and HONcode, the degree of agreement was quantified using Cohen’s weighted kappa coefficient. For data management and analysis, the above-mentioned statistical package R, version 3.6.3 [23], was used. *p*-values < 0.05 were considered significant. In view of the exploratory nature of the data analysis, no alpha-adjustment was performed.

## 3. Results

With regard to relevant content, i.e., non-commercial websites, search queries A (*n* = 103) and B (*n* = 120) had six identical hits, A and C (*n* = 81) and A and D (*n* = 128) only had one overlapping hit each. There were three identical hits between search queries B and C, seven for B and D, but none for C and D. Not a single hit appeared in every query. Therefore, the overlap can be considered relatively small, and thus, the search strategies can be regarded as largely complementary.

Some reputable domains were found in unexpected “backyards”. For example, the homepage of the “Arbeitsgemeinschaft Dermatologische Prävention” (ADP e.V.), which was responsible for the medical guideline on skin cancer prevention, was only found in 81st place in a search query (“B”) with its FAQ section [26]. In contrast, the Federal Office for Radiation Protection (BfS, [27]) was represented with three hits; in “A” on rank 37, in “B” on rank 3, and in “C” on rank 64. The German cancer aid (“Deutsche Krebshilfe”, [28]) was represented on rank 3 in search run “A”.

Across all search queries, the content completeness score, with a theoretical range of values from 0 to 43, only reached the median value of 10, first quartile (Q1) 6, third quartile (Q3) 14. The global quality score, on a scale of 0 to 10, was 3 on the median, Q1 2, Q3 4. The quality measured both by the content completeness score and the global quality score did not differ significantly between the four searches (*p* = 0.18 and *p* = 0.14, respectively); see Table 1. Meaningful visual material was found most frequently in search query C with 23.5%, while A with 11.8%, B with 11.7%, and above all, D with 7% offered useful visual material less frequently.

### 3.1. Search Query A: “Skin Cancer Sun Protection”

Of the 200 searches, three duplicates were found. Among the remaining 197 websites, 103 had relevant content and thus constitute the database for the following analyses. The amount of content information is shown in Table 1 (Investigator “C.E.”). Of the individual content items, skin cancer is mentioned in almost all hits, which is plausible given the search query. By contrast, fundamental aspects such as an appeal to avoid UV radiation were mentioned by about 72% of the web pages and avoidance of the midday sun was mentioned by 61%. The various sun protection measures follow a priority, with avoidance of strong sun exposure being the most important sun protection measure, followed by textile sun protection, and finally the use of sunscreens as a supplemental measure [8]. However, the correct supplemental use of sunscreens was represented by only a quarter of the websites. A recommendation to avoid sunbeds was given by 44% of the websites. At the end of Table 1, the distribution of both the content completeness score and the global quality score is shown.

**Figure 1:**
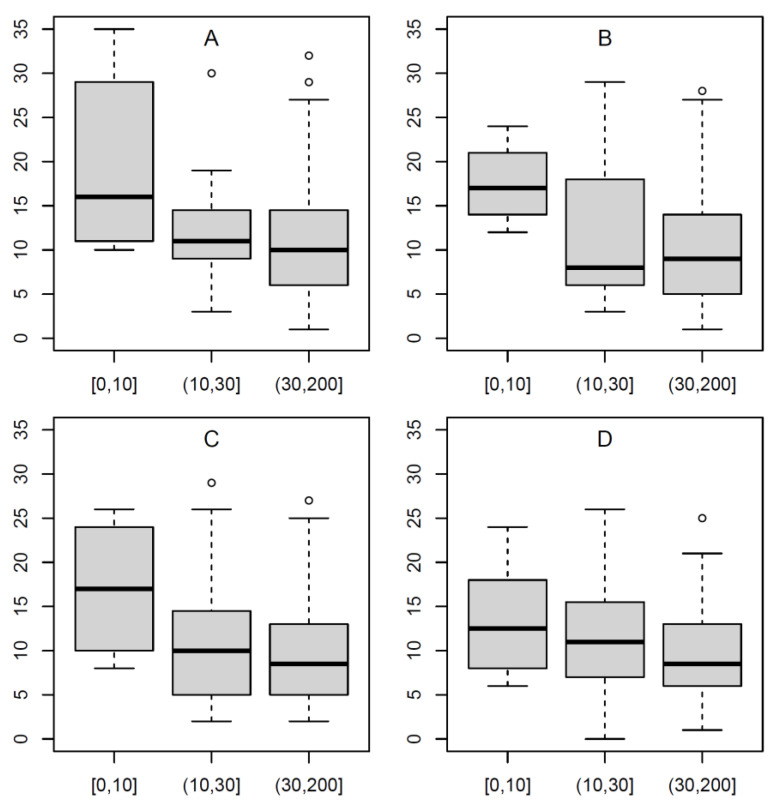
Distribution of the content completeness score for the four search queries (**A**–**D**) in three classes of the ranking of the query result: 1 to 10, 11 to 30, and 31 to 200, based on the initial, unfiltered search (definition of search query (**A**–**D**), see Section 2).

Moreover, the relationship of quality (in the above-mentioned sense of completeness and correctness of contents) and ranking of the website on the hit list was of interest. The present search query showed a significant, weak-negative correlation: *r* = −0.25, 95% CI: −0.43; −0.056. The content completeness scores in the three categories of ranks are shown in Figure 1A. It is evident that the “top hits” were of a clearly and significantly (*p* = 0.046) higher quality than the following hit pages.

Using the DISCERN score (Table 2), some aspects were largely adequately addressed, such as the objective of the information and balanced phrasing. On the other hand, important information was often missing—simple but fundamental aspects such as the time of writing, as well as indications of uncertainties or links to further information. This resulted in a moderate overall quality. The same applies to the HONcode (Table 3), where a good rating was often given for the intention of the website, data protection, and transparency, but there were common shortcomings in other areas such as underlying evidence or sources as well as lacking disclosure of funding or advertising.

### 3.2. Search Query B: “How to Protect Yourself from the Sun?”

In this search query, 120 of the 200 hits were further investigated as relevant. The completeness of the content aspects is shown in Table 1; compared to the first search query, reference to skin cancer is less common, that is, only presented in approximately 66% of the websites. In this search query, on the other hand, references to the wearing of suitable sunglasses were found most frequently; otherwise, there are no conspicuous differences in terms of the individual content criteria in relation to the other search queries. However, with about 14% mentions, the classification of sunscreens as the last instrument of UV prevention was mentioned quite rarely.

The results regarding DISCERN and HONcode in Table 2 and Table 3, respectively, resemble the pattern of search query A. The correlation between rank in the hit list and content quality was *r* = −0.26, 95% CI: −0.42; −0.078. The distribution of the content sum score in the three groups formed according to rank is shown in Figure 1B; the difference between the groups is also significant for this search query (*p* = 0.005).

### 3.3. Search Query C: “Sun Protection Children”

In the present search query, only 81 of the 200 websites were relevant in terms of content. A high number (*n* = 89) of the 119 excluded websites were commercial sites (companies, especially for sun protection products/sunscreens). The completeness of the content aspects is shown in Table 1. Reference to skin cancer was only made on 57% of the websites, while 93% made UV avoidance a topic. The rule of not exposing infants to the sun at all is plausibly found most frequently here, in 54% of websites.

The results concerning DISCERN and HONcode are shown in Table 2 and Table 3, respectively; the quality assessment was similar to that of the two search queries A and B. The correlation between rank in the hit list and quality of content was *r* = −0.22, 95% CI: −0.45; 0.031. The distribution of the content completeness score in the three groups according to rank is shown in Figure 1C—this was not significantly different (*p* = 0.19), at least partly owing to the relatively smaller size of this sub-sample.

### 3.4. Search Query D: “Preventing Sunburn”

In the search query “Preventing sunburn”, 128 of the 200 websites were relevant in terms of content. The completeness of the content aspects is shown in Table 1. Compared to the other search queries, the avoidance of midday sun is mentioned in 70.3%, and sunburn as a skin cancer risk factor is mentioned in almost 61% of websites; otherwise, there are no striking differences to the other search queries. Of note, although this search query explicitly addresses short-term UV damage, a correct classification of sunscreens as the last element in the ranking of sun protection measures is not mentioned at all.

The results regarding DISCERN and HONcode are found in Table 2 and Table 3, respectively, with a similar pattern as in search query C. The correlation between the rank in the hit list and the quality of the content was *r* = −0.2, 95% CI: −0.35; −0.027. The distribution of the content completeness score in the three groups according to rank is graphically illustrated in Figure 1D—this does not differ significantly between the three ranking categories (*p* = 0.061).

### 3.5. Relation between Generic and Content-Based Scores

**Figure 2:**
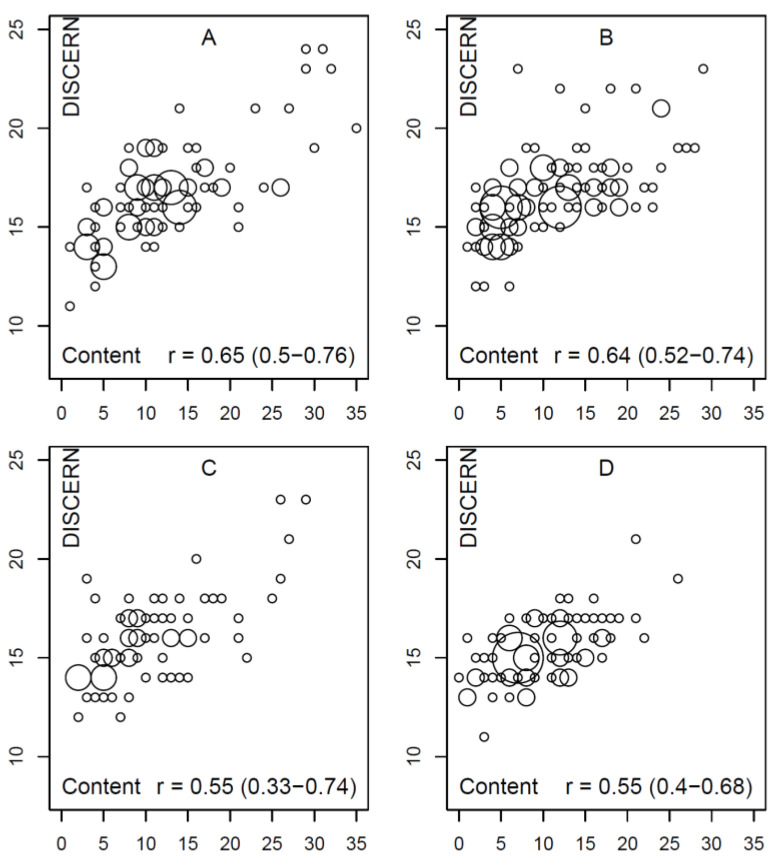
Correlation between the content completeness score (*x*-axis) and the DISCERN total score (*y*-axis) for the four search queries (**A**–**D**) as so-called “bubble plot”, where the size of the circle represents the frequency of co-occurrence of the score values (definition of search query (**A**–**D**), see Section 2).

The question of whether specific content quality or completeness and generic quality correlate was examined in relation to the DISCERN and the HONcode scores. The correlation between DISCERN and the content completeness score is shown in Figure 2 for the four search queries, including the respective correlation coefficients. With regard to the HONcode, a significantly weaker correlation was found (not shown graphically); the correlation for query A was r = 0.32, 95% CI: 0.1; 0.49, for B, it was r = 0.28, 95% CI: 0.082; 0.46, for C, it was r = 0.21, 95% CI: −0.036; 0.41, and for D, it was r = 0.075, 95% CI: −0.11; 0.26.

### 3.6. Reliability

To estimate the agreement between two investigators (“C.E.” and “W.U.”), the respective content completeness scores were correlated; the correlation coefficient according to Spearman was 0.75, 95% CI: 0.6; 0.87. With regard to the global quality score, the correlation was 0.69, 95% KI: 0.53; 0.81. The assessment according to DISCERN is summarized in Table 2, again as collected by “C.E.”. Concerning the eight individual ordinal items, the agreement quantified by Cohen’s Kappa coefficient, was in median 0.311, the correlation of the DISCERN score was 0.44, 95% CI: 0.24; 0.62. With regard to the HONcode, the results of which are presented in Table 3, the agreement, also quantified by Cohen’s kappa coefficient, was in the median 0.311, and the correlation of the total score of the eight HONcode items was 0.46, 95% CI: 0.28; 0.6.

## 4. Discussion

The present study has, to the best of our knowledge, for the first time systematically examined the quality of information retrievable from the internet on UV protection with the aim of preventing skin cancer or avoiding acute consequences (“Preventing sunburn”). The study was motivated by the observation that in an age- and gender-adjusted analysis, the use of the internet as a source of information on UV protection has been found to be associated with a rather poor knowledge [20]. Print and audiovisual media, or even lectures and individual consultations, i.e., the other sources of knowledge associated with a good level of knowledge in the above-mentioned study, are not equally accessible to a systematic investigation. For this reason, no comparisons can be made. However, even just examining the quality of information found on the internet reveals clear deficits.

Before discussing these deficits and possible consequences, the methodology of the study shall be discussed. From the technical point of view, it was important to avoid filtering the search results by sending the query from a “private” (i.e., non-academic) IP address range, and in each case with a new profile. In this way, referencing and thus distortions of the following search queries by previous queries can be prevented. Creating such a “tabula rasa” is certainly advisable, since otherwise, the results would be individually different due to the intransparent rules of Google™ and thus not comparable. From the large number of possible search terms and their combinations, four were selected for practical reasons. These appeared relevant and complementary on the basis of a preliminary survey. The complementarity was even confirmed beyond our expectations by the rather small overlap in the hits. However, the question of whether other search terms would have led to different results cannot be answered. Notwithstanding, we believe that a relatively comprehensive section was covered by terms that may be more aimed at older adults (“Skin cancer sun protection”), or terms that may apply to younger adults/parents (“Sun protection children”) or are otherwise of general interest. Independently, the relatively small overlap in the results of the search queries should be viewed critically: at least the high-quality offers should be identifiable by a large number of conceivable search terms, which is definitely not the case at present.

In order to estimate the reproducibility of the analysis, double evaluations were carried out for one of the search queries, which were mutually blinded. These showed a relatively good agreement with regard to the content of the evaluation with an *r* of 0.75; however, this was somewhat lower in the evaluation of the generic aspects. It should be noted that only the more general explanations on the DISCERN and HONcode websites were used and no separate definition of evaluation levels for the respective items was developed. Such an approach might have increased the level of agreement somewhat, without it being evident that the validity of the assessment would have increased in the same way. Thus, the results illustrate the difficulties of uniformly assessing “soft” criteria in particular. However, despite the above imperfections, we believe that our results can give a sufficiently valid impression.

Against the standard of the complete contents according to the medical guideline [8], the websites identified with the four search queries clearly offer incomplete information. Authoritative content from important and official institutions was sometimes found on the first hit page, but sometimes, it was also found on low ranks, making it unlikely that the content would actually be reached via a corresponding search. Irrespective of such outliers in the lower ranks, it can be seen that the quality and completeness of the content was highest on average for the first 10 websites (the first “hit page” on Google™). The click rates usually decrease significantly as the position of the hit decreases. The so-called “click-through rate” (CTR) indicates the percentage of how often a link on a page is called up in relation to how often the original page is called up. In this case, the original page is the Google™ hit list, and the clicked links are the listed search results. According to the “Google Organic CTR Study” [29], the CTR on the first five hits of the search is 67.6%, and on the first page with 10 hits, it is 71.3%; other such studies yielded similar results. Of course, it is possible—and less well studied—that users will still go on to the following hit pages and their search results (links) after clicking on one or more of the links on the first hit page; however, the importance of the ranking is clear. Even if the first ten hits were of better quality in relative terms than the subsequent ones, and in some cases even significantly so, the average quality observed in the top 10 field also appears to be a plausible explanation for the poor results reported at the beginning as motivation to study [19].

Our results are not surprising when compared to similar studies on other health-related topics, which have identified similar problems. Joury et al. showed with their study of websites showing information for patients with otitis media that there is also a lack of high-quality websites in this field, and only very few pages were written in a language that is accessible to a patient [30]. Johnson et al. reported in 2019 that online sources of information for patients with lymphoedema were only suitable for readers with a higher level of understanding, but were unsuitable and misleading for the general population [31]. Azer et al. also demonstrated a broad range of quality measured by DISCERN and HONcode in their study of information sites for patients with inflammatory bowel disease [32].

## 5. Conclusions

In order to better inform the population about UV avoidance and skin cancer prevention, the authoritative, official websites such as those of ADP, Deutsche Krebshilfe, or BfS (as examples in Germany) should be made more visible (identifiable). This could be done through relevant internet marketing strategies or through official agreements that ensure that defined, official sites of proven quality and topicality (such as those of the National Institute of Health in the USA) must be displayed by search engines in the “first 5 hits” area. Such an intervention in the “free market” will have to be discussed politically; however, in view of the current observation of limited quality and the earlier observation of poorer knowledge when this is researched on the internet, the need for action is evident. This notion is reinforced by the foreseeable further increase in the use of internet-based content and not least by the “infodemic” of misleading, false information observed in the context of the COVID-19 pandemic.

## Figures and Tables

**Table 1:** Content-related aspects of counselling with regard to UV protection for skin cancer prevention according to [8]: Absolute and relative frequencies of occurrences as well as, in the last two lines, the distribution of the content completeness score and the global quality score, respectively, for the four search queries A, B, C, and D (for the definition of A to D, see Methods). Q1, first quartile; Q3, third quartile.

Criterion	A: *n* (%)	B: *n* (%)	C: *n* (%)	D: *n* (%)
Number of relevant web pages	103	120	81	128
1 Addressing UV-related hazard				
1.1 Association with skin cancer	101 (98.1)	79 (65.8)	46 (56.8)	83 (64.8)
1.2 Leisure time exposure	27 (26.2)	16 (13.3)	7 (8.6)	2 (1.6)
1.3 Exposure in some occupations	16 (15.5)	15 (12.5)	2 (2.5)	3 (2.3)
1.4 Dependency on season and time of day	16 (15.5)	20 (16.7)	9 (11.1)	17 (13.3)
1.5 Dependency on weather (clouds)	18 (17.5)	6 (5)	7 (8.6)	7 (5.5)
1.6 Altitude (sea level, mountains)	25 (24.3)	25 (20.8)	5 (6.2)	17 (13.3)
1.7 Reflection by the ground (soil, sand, snow, water)	32 (31.1)	44 (36.7)	18 (22.2)	48 (37.5)
1.8 Exposure in the shade	26 (25.2)	30 (25)	24 (29.6)	40 (31.2)
1.9 Exposure also with overcast sky	34 (33)	31 (25.8)	21 (25.9)	29 (22.7)
1.10. What is UV (UV-A/-B/-C)	34 (33)	27 (22.5)	8 (9.9)	11 (8.6)
2 Motivation to change behavior: Avoid UV radiation	74 (71.8)	92 (76.7)	75 (92.6)	105 (82)
3 Avoid strong sun exposure				
3.1 Schedule outdoor activities to morning or evening	15 (14.6)	14 (11.7)	8 (9.9)	9 (7)
3.2 “Shadow rule”: Shadow shorter than object casting it = strong UV exposure	2 (1.9)	2 (1.7)	3 (3.7)	0 (0)
4 Avoid midday sun				
4.1 Mentioning of “Need to avoid”	63 (61.2)	67 (55.8)	50 (61.7)	90 (70.3)
4.2 Definition of the critical period (mostly 11:00–15:00)	46 (44.7)	48 (40)	42 (51.9)	55 (43)
5 Mentioning “Stay in the sun as brief as possible”	7 (6.8)	2 (1.7)	1 (1.2)	0 (0)
6 Seek shade				
6.1 Mentioning of “Seek shade”	31 (30.1)	37 (30.8)	36 (44.4)	36 (28.1)
6.2 Mentioning of diffused radiation e.g., on the beach or beneath umbrella	8 (7.8)	6 (5)	2 (2.5)	1 (0.8)
7. Avoid sunburn				
7.1 Mentioning of “Avoid sunburn”	23 (22.3)	18 (15)	15 (18.5)	48 (37.5)
7.2 Skin cancer risk is strongly increased by sunburns	48 (46.6)	45 (37.5)	35 (43.2)	78 (60.9)
8 UV-Index (UVI)				
8.1 Explanation of the UVI	19 (18.4)	17 (14.2)	9 (11.1)	10 (7.8)
8.2 Sun protection related to UVI recommended	11 (10.7)	10 (8.3)	3 (3.7)	10 (7.8)
8.3 Explanation where UVI information is published	23 (22.3)	16 (13.3)	8 (9.9)	11 (8.6)
9 Mentioning of “Slow adaptation of the skin to sun exposure”	14 (13.6)	23 (19.2)	5 (6.2)	24 (18.8)
10 Wearing of sun-protective clothing				
11.1 Sun protection factor mentioned	45 (43.7)	72 (60)	44 (54.3)	34 (26.6)
11.2 Long-sleeved clothing	27 (26.2)	23 (19.2)	17 (21)	22 (17.2)
11.3 Special beach/swimming garments	10 (9.7)	12 (10)	16 (19.8)	3 (2.3)
12 Sunscreens				
12.1 Correct mentioning as “Last/subsidiary measure”	25 (24.3)	17 (14.2)	11 (13.6)	0 (0)
12.2 Mentioning of adequate sun protection factor	70 (68)	81 (67.5)	52 (64.2)	96 (75)
12.3 Application of a sufficient quantity (2 mg/cm^2^)	46 (44.7)	44 (36.7)	21 (25.9)	27 (21.1)
12.4 Application before sun exposure	38 (36.9)	41 (34.2)	32 (39.5)	55 (43)
12.5 Repeat application e.g., after swimming (not providing longer protection)	53 (51.5)	55 (45.8)	52 (64.2)	61 (47.7)
12.6 Waterproof sunscreens recommended	19 (18.4)	27 (22.5)	22 (27.2)	24 (18.8)
13 Sensitivity against sunlight				
13.1 Mentioning of Fitzpatrick skin types or “Celtic type”	36 (35)	42 (35)	16 (19.8)	60 (46.9)
13.2 Counselling depending on skin type of patient	24 (23.3)	12 (10)	1 (1.2)	15 (11.7)
14 Presentation of Fitzpatrick skin type or “Celtic type”	28 (27.2)	23 (19.2)	9 (11.1)	26 (20.3)
15 Reference to drug information on possible adverse reactions to sun	13 (12.6)	17 (14.2)	6 (7.4)	24 (18.8)
16 Children				
16.1 Children should not sustain sunburn	17 (16.5)	18 (15)	15 (18.5)	9 (7)
16.2 Infants should not be exposed to direct sunlight	30 (29.1)	34 (28.3)	44 (54.3)	33 (25.8)
17 Avoid sunbeds: Any justification/detail	45 (43.7)	21 (17.5)	4 (4.9)	18 (14.1)
18. Sunglasses				
18.1 Reference to sufficiently UV-protective quality	32 (31.1)	48 (40)	30 (37)	25 (19.5)
18.2 European norm EN 1836 mentioned	1 (1)	3 (2.5)	0 (0)	0 (0)
18.3 Glare categories mentioned	3 (2.9)	10 (8.3)	2 (2.5)	0 (0)
Content completeness score (Median; Q1–Q3)	11 (8–15)	10 (5–16)	9 (5–13)	9 (6–13)
Global quality score (Median; Q1–Q3)	3 (2–5)	3 (1–4)	3 (2–4)	3 (2–3)

**Table 2:** Absolute (*n*) and relative (%) frequencies of DISCERN single items results; in the last row, distribution of the DISCERN total score of web pages devoted to UV protection for skin cancer prevention for the search queries A, B, C, and D, respectively (definition of search query A to D, see Methods). Q1, first quartile; Q3, third quartile.

Criterion		A: *n* (%)	B: *n* (%)	C: *n* (%)	D: *n* (%)
Defined objectives	Yes	97 (95.1)	112 (93.3)	47 (58)	56 (43.8)
	Partly	5 (4.9)	7 (5.8)	24 (29.6)	30 (23.4)
	No	0 (0)	1 (0.8)	10 (12.3)	42 (32.8)
Defined objectives have been met	Yes	82 (80.4)	93 (78.2)	38 (53.5)	50 (58.1)
	Partly	17 (16.7)	25 (21)	31 (43.7)	35 (40.7)
	No	3 (2.9)	1 (0.8)	2 (2.8)	1 (1.2)
Significant publication	Yes	58 (56.9)	70 (58.3)	51 (63)	72 (56.2)
	Partly	36 (35.3)	34 (28.3)	24 (29.6)	46 (35.9)
	No	8 (7.8)	16 (13.3)	6 (7.4)	10 (7.8)
Transparent information sources	Yes	12 (11.8)	9 (7.5)	8 (9.9)	12 (9.4)
	Partly	17 (16.7)	22 (18.3)	13 (16)	8 (6.2)
	No	73 (71.6)	89 (74.2)	60 (74.1)	108 (84.4)
Indication of date of publication	Yes	4 (3.9)	3 (2.5)	2 (2.5)	3 (2.3)
	Partly	59 (57.8)	82 (68.3)	38 (46.9)	75 (58.6)
	No	39 (38.2)	35 (29.2)	41 (50.6)	50 (39.1)
Well-balanced and objective presentation	Yes	90 (88.2)	114 (95)	73 (90.1)	115 (89.8)
	Partly	12 (11.8)	6 (5)	8 (9.9)	12 (9.4)
	No	0 (0)	0 (0)	0 (0)	1 (0.8)
Detailed information on supplemental help and further information	Yes	7 (6.9)	5 (4.2)	4 (4.9)	2 (1.6)
	Partly	10 (9.8)	6 (5)	9 (11.1)	3 (2.3)
	No	85 (83.3)	109 (90.8)	68 (84)	123 (96.1)
Areas of uncertainty have been mentioned	Yes	7 (6.9)	13 (10.8)	5 (6.2)	0 (0)
	Partly	6 (5.9)	10 (8.3)	4 (4.9)	1 (0.8)
	No	89 (87.3)	97 (80.8)	72 (88.9)	127 (99.2)
DISCERN total Median (Q1–Q3)		16.5 (15–17)	16 (15.5–18)	16 (14–17)	15 (14–16)

**Table 3:** Absolute (*n*) and relative (%) frequencies of HONcode single items results; in the last row, distribution of the HONcode total score of web pages devoted to UV protection for skin cancer prevention for the search queries A, B, C, and D, respectively (definition of search query A to D, see Methods). Q1, first quartile; Q3, third quartile.

Criterion		A: *n* (%)	B: *n* (%)	C: *n* (%)	D: *n* (%)
Expertise	Yes	31 (30.4)	14 (11.7)	10 (12.3)	16 (12.5)
	Partly	11 (10.8)	14 (11.7)	11 (13.6)	2 (1.6)
	No	60 (58.8)	92 (76.7)	60 (74.1)	110 (85.9)
Complementarity (intention of the website)	Yes	94 (92.2)	119 (99.2)	78 (96.3)	120 (93.8)
	Partly	8 (7.8)	1 (0.8)	3 (3.7)	8 (6.2)
	No	0 (0)	0 (0)	0 (0)	0 (0)
Data protection	Yes	85 (83.3)	111 (92.5)	76 (93.8)	118 (92.2)
	Partly	0 (0)	2 (1.7)	3 (3.7)	2 (1.6)
	No	17 (16.7)	7 (5.8)	2 (2.5)	8 (6.2)
Sources defined	Yes	10 (9.8)	12 (10)	8 (9.9)	16 (12.5)
	Partly	8 (7.8)	15 (12.5)	12 (14.8)	7 (5.5)
	No	84 (82.4)	93 (77.5)	61 (75.3)	105 (82)
Evidence available	Yes	9 (8.8)	2 (1.7)	3 (3.7)	1 (0.8)
	Partly	4 (3.9)	9 (7.5)	7 (8.6)	4 (3.1)
	No	89 (87.3)	109 (90.8)	71 (87.7)	123 (96.1)
Transparency	Yes	97 (95.1)	117 (97.5)	80 (98.8)	127 (99.2)
	Partly	1 (1)	0 (0)	0 (0)	0 (0)
	No	4 (3.9)	3 (2.5)	1 (1.2)	1 (0.8)
Declaration of funding	Yes	17 (16.7)	14 (11.7)	9 (11.1)	8 (6.2)
	Partly	4 (3.9)	7 (5.8)	1 (1.2)	2 (1.6)
	No	81 (79.4)	99 (82.5)	71 (87.7)	118 (92.2)
Advertising policy	Yes	32 (31.4)	31 (25.8)	14 (17.3)	25 (19.5)
	Partly	31 (30.4)	36 (30)	15 (18.5)	17 (13.3)
	No	39 (38.2)	53 (44.2)	52 (64.2)	86 (67.2)
HONcode total Median (Q1–Q3)		16.5 (15–17)	15 (14–17)	15 (14–17)	14 (14–16)

## Data Availability

The data presented in this study are available upon request.
